# Low frequency ultrasonic debridement: a clinical experience

**DOI:** 10.1186/1757-1146-4-S1-P41

**Published:** 2011-05-20

**Authors:** Lucia Michailidis, Gillian Butcher, Annette Davis

**Affiliations:** 1 Podiatrist, Southern Health, Victoria, 3169, Australia; 2 Acute Podiatry Services Manager, Southern Health, Victoria, 3169, Australia; 3 Sub-acute Podiatry Services Manager, Southern Health, Victoria, 3169, Australia

## 

Low Frequency Ultrasonic Debridement (LFUD) provided by the Sonoca 185 is a new method of debriding wounds that is less traumatic, less painful and can achieve faster healing rates. It is bactericidal and enables many debridement treatments to be undertaken by a podiatrist or wound consultant in their smaller clinical setting. Due to the many properties of LFUD the clinical indications for use are vast including but not limited to chronic ulcers, diabetes foot ulcers, leg ulcers, pressure ulcers, infected ulcers and dehisced wounds. As with all treatment methods there are some contraindications to use of LFUD treatment which include but not limited to those patients with vascular abnormalities, those patients with haemorrhagic conditions, over exposed malignancies and tissue previously treated with radiation, deep x-ray or irradiation. This technology is new within Victoria and to the departments of podiatry and wound care specialists. Being so, all staff directly involved with the daily clinical use of LFUD for wound management were up skilled and had to pass competencies to use the treatment modality. In addition, clinical pathways and protocols were developed in order to ensure best patient care was practised. This presentation will give an overview of the process involved in using LFUD including (i) patient selection, (ii) clinical set up, and (iii) considerations during treatment. By discussing and evaluating clinical case studies on the use of LFUD I will further demonstrate its benefits in wounds healing and provide my opinion and experience on the ease of using this effective debriding agent.

